# Review of Salivary Gland Neoplasms

**DOI:** 10.5402/2012/872982

**Published:** 2012-02-16

**Authors:** Victor Shing Howe To, Jimmy Yu Wai Chan, Raymond K. Y. Tsang, William I. Wei

**Affiliations:** Division of Otorhinolaryngology, Head and Neck Surgery, Department of Surgery, The University of Hong Kong, Queen Mary Hospital, Hong Kong

## Abstract

Salivary gland tumours most often present as painless enlarging masses. Most are located in the parotid glands and most are benign. The principal hurdle in their management lies in the difficulty in distinguishing benign from malignant tumours. Investigations such as fine needle aspiration cytology and MRI scans provide some useful information, but most cases will require surgical excision as a means of coming to a definitive diagnosis. Benign tumours and early low-grade malignancies can be adequately treated with surgery alone, while more advanced and high-grade tumours with regional lymph node metastasis will require postoperative radiotherapy. The role of chemotherapy remains largely palliative. This paper highlights some of the more important aspects in the management of salivary gland tumours.

## 1. Introduction

Salivary gland tumours are rare and most cases are referred to the head and neck clinic. The majority of these neoplasms are benign and only 20% are malignant. The annual incidence of salivary gland cancers ranges from 0.5 to 2 per 100,000 in different parts of the world, with the highest incidence occurring in Croatia [1]. In the United states, there is a rise in the incidence of salivary gland cancers; this group accounted for 6.3% of all head and neck cancers in 1974–1976, as compared to 8.1% in 1998-1999 [2]. The sex distribution for salivary gland cancers is equal, and the majority of the cases arise in the sixth decade [3]. Tumours can occur in both the major and minor salivary glands. 80% of major salivary gland tumours occur in the parotid glands, while most minor salivary tumours are located in the palate [4]. As a general rule in clinical practice, the smaller the salivary gland is, the more likely the tumour is malignant. In the parotid glands, 20–25% of the tumours are malignant. This rises to 40% for the submandibular glands, and more than 90% of sublingual gland tumours are malignant [5, 6].

The etiological agents of salivary gland cancers remain unclear. Whilst most other head and neck cancers are strongly related to smoking and drinking, these do not play a role in the salivary glands. Some studies have found that a diet rich in vitamin C and low in cholesterol may be effective in preventing salivary gland cancer [7]. On the other hand, possible risk factors include therapeutic radiation for other head and neck cancers, occupational exposures in rubber manufacturing and woodworking, and also employment at hairdressers or beauty shops [8, 9]. History of previous cancers, related to Epstein-Barr virus, immunosuppression, and radiation were also associated with an increased risk of salivary gland cancer. In a Swedish study, the risk of salivary gland cancer was increased 4 fold in Hodgkin's lymphoma patients [10]. HIV infection was also found to increase the risk of salivary gland cancers [11].

Salivary gland tumours in the parotid or submandibular glands usually present as an enlarging mass. This may be associated with neurological symptoms such as facial nerve paralysis or pain if the tumour is malignant. Minor salivary gland tumours present as a submucosal intraoral mass which subsequently ulcerates. Clinical features suspicious for malignancy include ipsilateral facial nerve palsy, sudden tumour growth, pain, tumour fixation to the overlying skin or underlying muscle, and cervical lymphadenopathy.

## 2. Histology

In both the major and minor salivary glands, the commonest type of benign tumour is pleomorphic adenoma. Pleomorphic adenoma, also called benign mixed tumour, can occur in the deep lobe of the parotid gland with extension into the parapharyngeal space, which makes it the commonest type of parapharyngeal space tumour constituting 40% of tumours there [12]. Pleomorphic adenomas need to be managed diligently for its capacity to recur and to undergo malignant transformation. Capsular rupture and subsequent tumour spillage during excision is the most important risk factor for recurrence. The risk of malignant change has been report to be up to 10%. Features predictive of malignant change include age, tumour size, a long history of the mass, submandibular location, and the presence of hyalinized stroma [13].

For malignant salivary tumour, the commonest type overall is mucoepidermoid carcinoma. However, if we look at each salivary gland in turn, several large series have shown that mucoepidermoid carcinoma is only the commonest cancer in the parotid glands, comprising around 33% [14, 15]. Adenoid cystic carcinoma is the commonest cancer in the submandibular and minor salivary glands, making up 42–49% [14, 15]. A review of 45 parotid cancer patients treated at the author's institute from 2000 to 2010 also shows mucoepidermoid carcinoma to be the commonest type (Table 1).

Mucoepidermoid carcinoma is a distinct histological type with a wide variation in clinical behavior. As such, histological grading is particularly important in predicting the prognosis of this group of patients. The tumour is classified as of low, intermediate, or high grade based on various histopathological criteria such as intracystic component, neural invasion, necrosis, mitosis, lymphovascular invasion, and bone invasion [16]. The five-year disease-free survival for low-grade tumours is reported to be 80–95%, while that for high-grade tumours is 30–50% [17]. Adenoid cystic carcinoma is a more indolent tumour with a prolonged disease history. These tumours metastasize quite early because of their propensity for perineural invasion and hematological spread, with the lungs being the most common site. Even with distant metastases, however, patients can survive for 15 to 20 years. A review of 50 patients with adenoid cystic carcinoma actually showed that the location of salivary gland involved, tumour stage, node positivity, and perineural invasion did not affect overall survival or local control [18]. The overall survival rates are 50–90% at five years and 30–67% at 10 years [19]. Acinic cell carcinoma is also considered to be a low-grade tumour which seldom invades the facial nerve. Regional recurrences occur late in this tumour but 16–20% of patients eventually die of this cancer [14].

## 3. Investigations

**Figure 1:**
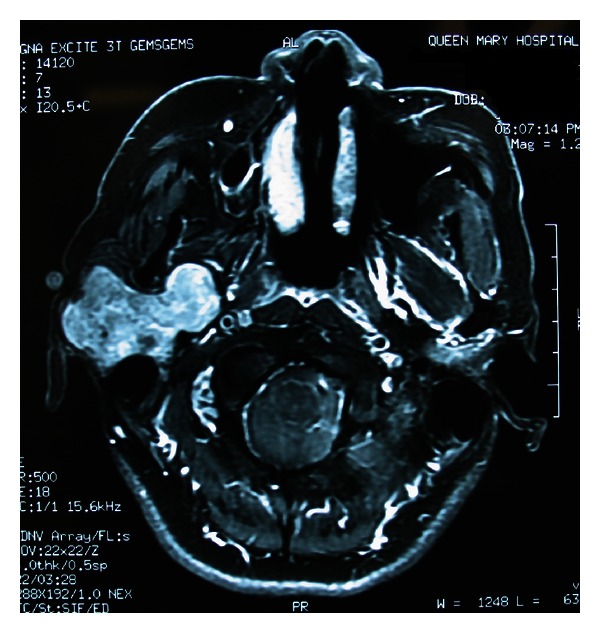
MRI showing right parotid tumour with extension into the deep lobe.

Radiological investigations are frequently employed in the workup of a salivary gland tumour. It is used to delineate the tumour location such as intraglandular or extraglandular and whether it is in the superficial or deep lobe of the parotid gland, to detect malignant features, to define local extension and invasion of surrounding tissues, and to detect regional nodal and systemic metastases. Ultrasound is the preferred tool for initial assessment of tumours in the superficial parotid and submandibular gland. Ultrasound imaging resolution of these superficial structures is excellent and it does not carry any risk of radiation. It can also be employed to guide the needle when carrying out fine needle aspiration cytology (FNAC) to reduce sampling error. For tumours located deep in the parotid gland or in the minor salivary glands, CT and MRI are more useful. Comparing the two modalities, MRI has an edge over CT in predicting malignancy and is also more sensitive in picking up small tumours. Benign tumours generally show high signal on T2-weighted scans, while malignant lesions usually show intermediate to low signal. MRI is more useful in detecting deep lobe extension (Figure 1), marrow infiltration, and perineural spread and involvement of the facial nerve. MR spectroscopy is employed to differentiate malignant and benign salivary gland tumours and also to distinguish Warthin's tumour from pleomorphic adenoma [20]. The role of CT is usually limited to evaluation of cortical bone involvement and the concurrent presence of calculus disease. The role of positron emission tomography (PET) in salivary gland disease is still being evaluated. Characteristically, Warthin's tumours and oncocytomas show a strong uptake of technetium pertechnetate.

Fine needle aspiration cytology (FNAC) is another investigation frequently used. The diagnostic yield of FNAC can be quite good with large study series showing sensitivity up to 85% and specificity up to 99% [21]. However, reports of other studies are far from ideal, and thus controversy as to the usefulness of FNAC still persists. It is safe to say that the role of FNAC mainly lies in preoperative patient counseling and surgical planning. In the case of a malignant FNAC result, the patient can be better prepared in terms of the extent of surgery, higher potential complications, and need for neck dissection or postoperative radiotherapy.

## 4. Staging

From 2010 onwards, salivary gland cancers are staged according to the Seventh Edition of the American Joint Committee on Cancer (AJCC) Cancer Staging Manual. The primary tumour (T) is staged according to size, extraparenchymal extension, and direct invasion. Regional lymph node (N) staging is dependent on the size and location of the metastatic lymph nodes. The detection of distant metastasis is designated as M1. The overall cancer stage is derived from the combination of these three factors.

## 5. Treatment

**Figure 2:**
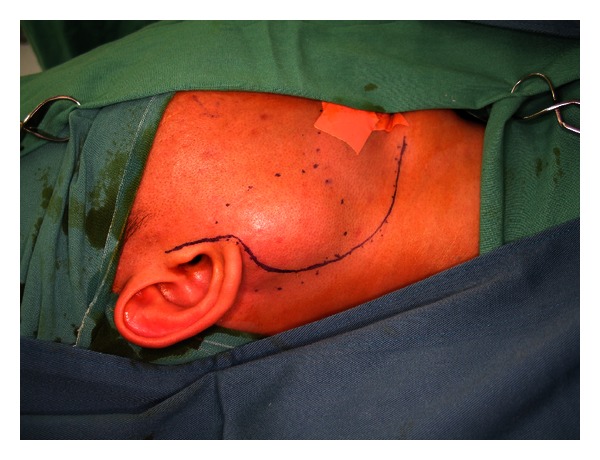
Incision for parotidectomy.

**Figure 3:**
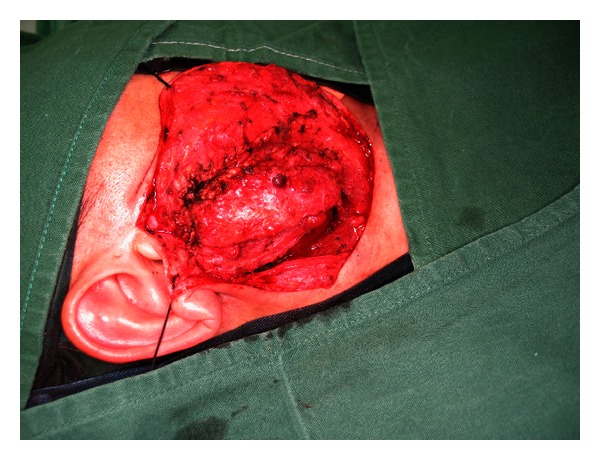
Skin flap raised.

**Figure 4:**
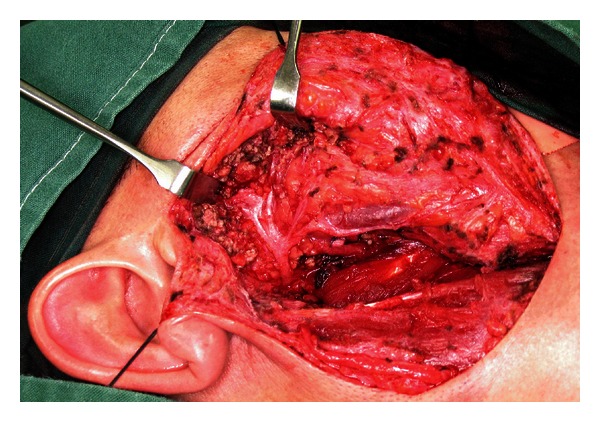
All branches of facial nerve dissected.

Surgery is the mainstay of treatment for salivary gland tumours. In the case of parotid gland tumours, superficial parotidectomy with facial nerve dissection and preservation is the standard diagnostic procedure. This operation is also therapeutic in cases of benign or small malignant tumours limited to the superficial lobe of the gland. Studies in the past have found enucleation of benign tumours to be associated with an unacceptably high chance of tumour recurrence especially for pleomorphic adenomas [22]. However, several recent studies have suggested that a more limited resection in the form of extracapsular dissection where the pleomorphic adenoma is resected only with a cuff of normal glandular tissue offers better postoperative cosmetic result and lower incidence of Frey's syndrome without an increase in the incidence of tumour recurrence and facial nerve injury [23–25]. Recurrence rates with either approach are in the order of 1–3% in the literature [26]. Whether a superficial or limited partial parotidectomy is carried out, the surgical approach is similar. A modified Blair's incision which begins just anterior to the tragus and then curves posteriorly towards the mastoid process and then gently turns anteriorly and inferiorly towards a neck skin crease is used (Figure 2). A skin flap is then raised anteriorly in the relatively avascular plane of the superficial musculoaponeurotic system (SMAS) (Figure 3). The parotid gland is dissected free from the tragus and sternocleidomastoid muscle in order to expose the main trunk of the facial nerve as it exits the stylomastoid foramen. The most consistent landmark used in its identification is the tympanomastoid suture which can be palpated easily. The rest of the operation involves dissection of individual branches of the facial nerve which will end up with removal of the superficial lobe of the parotid gland (Figure 4).

If the tumour involves the deep lobe of the parotid gland, a total parotidectomy is the procedure of choice to achieve adequate tumour clearance. This entails full dissection of all branches of the facial nerve from the superficial lobe, followed by delivery of the deep lobe from underneath the nerve. Total parotidectomy with facial nerve preservation is also indicated for high-grade malignancies and T3-4 cancers. In these cases, preoperative MRI is very important in order to delineate the proximity of the tumour to the internal carotid artery and skull base [27]. Radical parotidectomy, in which the facial nerve is sacrificed, is only necessary if the tumour is enveloping or infiltrating the facial nerve. In such cases, there is often a degree of facial nerve paralysis evident preoperatively. After facial nerve resection, the nerve is most often repaired with an interposition graft from another nerve. Even in experienced hands, the facial nerve function rarely recovers fully and only House-Brackmann Grade III can be achieved.

**Figure 5:**
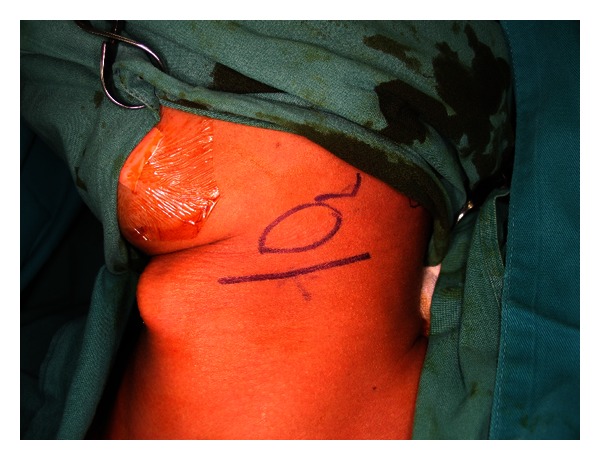
Incision for submandibular gland excision.

**Figure 6:**
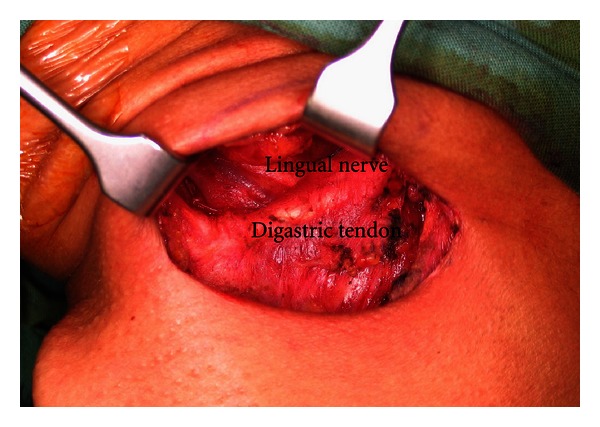
Gland bed after submandibular gland removal.

When dealing with submandibular gland tumours, complete excision of the gland is adequate treatment if the lesion is small, limited to the gland parenchyma, and also of benign or low-grade malignant nature. More extensive tumours require excision of the gland bed and also adjacent soft tissues similar to a supraomohyoid neck dissection [22]. The standard approach is via a transverse incision placed two finger-breadths below the mandible (Figure 5) and then elevation of a subplatysmal flap with careful identification and protection of the marginal mandibular branch of the facial nerve. The facial vessels are ligated and the gland is dissected free from the digastric and mylohyoid muscles while safeguarding the hypoglossal and lingual nerves. The gland is finally excised after ligation of the Wharton's duct (Figure 6).

Management of the clinically N0 neck in salivary gland cancer remains a controversial topic. Studies on the long-term efficacy of various approaches in dealing with N0 neck are limited. Some advocate neck dissection for every salivary gland cancer patient, while others choose selective neck dissection or irradiation of the neck for high-risk cancers only [28–30]. Because of the small size of these studies, no single approach has been shown to be superior. Overall, the risk of regional lymph node metastasis in salivary gland cancer is low compared to other head and neck cancers and range from 14–20% [31]. High-risk factors include high-grade and advanced T-stage tumours, tumours with extracapsular extension, and presence of preoperative facial paralysis [32]. As such, elective selective neck dissection may be recommended in these patients. Elective neck dissection should also be performed if resection of the primary tumour is aided by removal of the surrounding lymph nodes. An exception to this is in the case of adenoid cystic carcinoma in which the chance of occult lymph node metastasis is low and elective neck dissection is unlikely to offer any additional benefit [28]. Selective neck dissection for parotid gland cancer should include levels IB, II, III, IV, and VA, while that for submandibular gland cancer should include levels I, II, and III. For patients with proven nodal metastasis, a full radical neck dissection should be carried out.

Postoperative radiotherapy has proven benefit in salivary gland cancer patients at high risk of locoregional recurrence. Several studies have identified important prognostic factors (Table 2) and postoperative radiotherapy is recommended [14, 33].

The different types of radiotherapy for salivary gland cancer include electrons, electron/photon mix, protons, and neutrons. Neutron beam radiation therapy has been shown to be more effective in certain tumour histology especially adenoid cystic carcinoma [34]. Intensity modulated radiation therapy is a popular technique since it offers conformal radiation producing steep dose gradient which allows for excellent tumour coverage while reducing dose to surrounding normal tissues. The usual postoperative radiotherapy dosage ranges from 60 to 70 Gy in 2 Gy daily fractions [27].

The role of chemotherapy remains to be largely palliative in treating metastatic, recurrent, or unresectable salivary gland cancers. Due to the paucity of these patients, randomized Phase III studies on the use of chemotherapy are lacking. Cisplatin is currently the single agent most often used. Combination therapies with cisplatin, 5-fluorouracil, cyclophosphamide, and doxorubicin have also been studied, but the response rates are quite variable due to the small number of patients enrolled [35, 36]. More recently, targeted therapies have also been studied, and the targets include epidermal growth factor receptor (EGFR), HER2 protooncogene, c-kit protooncogene, and vascular endothelial growth factor (VEGF). Thus far, no target therapy agent has been found to be effective [14].

## 6. Conclusion

Salivary gland tumours are best managed in specialized head and neck clinics because of their rarity and need for thorough workup by a multidisciplinary team of specialists. Surgery forms the keystone of their management since it serves both diagnostic and therapeutic purposes. The most important step is in the surgical planning and preoperative counseling. Large or deep parotid lobe tumours, and those suspected to be malignant, should be imaged preferably with MRI in order to decide on the expected extent of resection and to determine the potential risk to the facial nerve. The whole surgical exercise in removing a parotid tumour revolves around careful identification and preservation of the facial nerve. Injury to the facial nerve causes significant morbidity to the quality of life of the patients. Elective selective neck dissection in clinically N0 salivary gland cancer is recommended when the primary tumour exhibits high-risk features. On the same note, postoperative radiotherapy is useful for tumours at risk of locoregional recurrence. The role of chemotherapy is as yet not well established and investigations of targeted therapy are still at an early stage.

## Figures and Tables

**Table 1:** Distribution of histological types of parotid cancers treated in QMH from 2000–2010.

Histology	Number (%) (total = 45)
Mucoepidermoid carcinoma	11 (24.4%)
Acinic cell carcinoma	9 (20%)
Salivary duct carcinoma	8 (17.7%)
Carcinoma expleomorphic	4 (8.9%)
Adenoid cystic carcinoma	3 (6.7%)
Others	10 (22.3%)

**Table 2:** Indications for postoperative radiotherapy.

Risk factors for locoregional recurrence in salivary gland cancers	
Close or positive resection margins	
High-grade or undifferentiated tumours	
Perineural invasion	
Skin or bone invasion	
Advanced disease involving facial nerve or deep lobe	
Lymph node metastases	
Tumour spillage during operation	
Unresectable or recurrent tumour	
